# Understanding General Somatic Symptom Burden: Insights from a Systematic Review of Factor Analyses Pertaining to the Patient Health Questionnaire 15 (PHQ-15) and Somatic Symptom Scale 8 (SSS-8)

**DOI:** 10.1007/s12529-025-10365-y

**Published:** 2025-04-23

**Authors:** Jonna Hybelius, Amanda Kosic, Sigrid Salomonsson, Caroline Wachtler, John Wallert, Steven Nordin, Erland Axelsson

**Affiliations:** 1 Division of Family Medicine and Primary Care, Department of Neurobiology, Care Sciences and Society, Karolinska Institutet, Stockholm, Sweden; 2 Liljeholmen University Primary Health Care Centre, Academic Primary Health Care Centre, Region Stockholm, Stockholm, Sweden; 3 School of Law, Psychology and Social Work, Örebro University, Örebro, Sweden; 4 Centre for Psychiatry Research, Department of Clinical Neuroscience, Karolinska Institutet, & Stockholm Health Care Services, Region Stockholm, Stockholm, Sweden; 5 Department of Psychology, Umeå University, Umeå, Sweden

**Keywords:** Factor analysis, Patient health questionnaire, Persistent physical symptoms, Somatic symptom burden, Systematic review

## Abstract

**Background:**

Factor analyses have indicated that somatic symptom burden can be separated into local symptom domain factors (e.g., cardiopulmonary, fatigue, gastrointestinal, pain) and a general propensity toward being symptomatic. This study aimed to determine what specific physical symptoms, and correlates, that are most strongly associated with this general factor.

**Method:**

A systematic review was based on factor analyses of the Patient Health Questionnaire 15 (PHQ-15) and Somatic Symptom Scale 8 (SSS-8).

**Results:**

There was heterogeneity in the included studies, in terms of the exact specification of the factor structure, and to some extent regarding item inclusion for factor analysis. Among 11 analyses of the PHQ-15, the highest mean and median factor loadings on the general symptom burden factor were seen for fatigue (*M* = 0.65) followed by dizziness (0.63). Among three analyses of the SSS-8, the mean was highest for chest pain and shortness of breath (0.69), followed by fatigue (0.62). The PHQ-15 general factor exhibited variable, but usually moderate to strong, associations with anxiety and depression symptoms, health anxiety, somatosensory amplification, and functional somatic syndromes.

**Conclusions:**

Cardiopulmonary symptoms and fatigue appear to be especially closely associated with general somatic symptom burden. The close associations between this general factor and indicators of poor mental health and functional somatic syndromes allow for numerous interpretations; both causal and due to overlapping definitions.

**Supplementary Information:**

The online version contains supplementary material available at 10.1007/s12529-025-10365-y.

## Introduction

Persistent physical symptoms — such as cardiopulmonary, fatigue, gastrointestinal, and pain complaints — are reported by a large and heterogeneous patient group. This clinical problem is associated with substantial challenges for the individual, healthcare, and society at large [1]. Merely the subset of persistent physical symptoms that lack a clear medical explanation have socioeconomic repercussions comparable to those of major depression or anxiety disorders [2]. A recurrent discussion in this research field is under what circumstances it is valid and useful to emphasize overlaps in the experience of common persistent symptoms, such as pain, fatigue, and muscle tension, rather than to manage each such symptom, or domain of symptoms, in isolation [3, 4]. Among clinicians and researchers interested in general somatic symptom burden, there is also little agreement over theoretical and diagnostic terms [e.g., 5–7]. A central research question is therefore to what extent, how, and why common persistent physical symptoms occur together.

Structural equation modeling strains of factor analysis present a powerful method of studying the distinct contributions of general and domain-specific factors to specific physical symptoms [8]. We recently completed a comprehensive systematic review of the measurement properties of two widespread patient-reported instruments measuring somatic symptom burden: the Patient Health Questionnaire 15 (PHQ- 15) and the Somatic Symptom Scale 8 (SSS- 8) [9]. PHQ- 15 comprises 15 items and focuses on the past 4 weeks [10], while SSS- 8 is an abbreviated version that consists of eight items focusing on the past week [11]. As part of the systematic review, we tabulated key outcomes from factor analyses, and found that when the method of analysis allowed this to be captured, both the PHQ- 15 and SSS- 8 appeared to exhibit a multifactorial structure. Typically, this factor solution included both a factor reflecting the general tendency of being symptomatic — that is, a general somatic symptom burden factor on which all items loaded either directly or indirectly — and symptom-specific domains (factors) that were distinct for cardiopulmonary, fatigue, gastrointestinal, and pain symptoms. However, as highlighted by Luciano et al. [12], the fact that a general factor emerges in a number of factor analyses does not necessarily mean that these general factors are sufficiently similar to be interpreted in the same way, rather than merely reflecting sample-specific random error unaccounted for by the domain-specific factors. In order to evaluate to what degree general factors are comparable over studies and settings, it is important to study the variance in factor loadings, and the relationship with other key variables of interest.

In this secondary study to the systematic review, we aimed to evaluate to what degree there is consistency in what somatic symptoms measured by the PHQ- 15 and SSS- 8 load strongest onto the general somatic symptom burden factor. Should a consistent pattern emerge, this would speak in favor of the validity of interpreting the general factor in a similar way across studies. Further, specific symptoms with a high loading on the general factor could be particularly indicative of a tendency to experience many physical symptoms in general, which could be clinically useful. In line with previous findings [13–16], we hypothesized that symptoms with especially pronounced loadings on the general factor would be cardiopulmonary symptoms and fatigue. We also aimed to review all reported clinical correlates with the general factor.

## Methods

### Design

This was a secondary study based on a comprehensive systematic review of the measurement properties of the PHQ- 15 and the SSS- 8 [9]. The study was based at the Department of Neurobiology, Care Sciences and Society, Karolinska Institutet, and Liljeholmen University Primary Health Care Centre, Stockholm, Sweden. Results are reported in accordance with the Preferred Reporting Items for Systematic Reviews and Meta-Analyses (PRISMA) 2020 [17].

### Searches and Eligibility Criteria

This systematic review was based on a combination of three searches: First, we searched the Medline, PsycINFO, and Web of Science by combining search terms for a focus on measurement properties (such as “psychometric*”, “valid*”, and “factor analy*”) with search terms for PHQ- 15 and SSS- 8 (such as “patient health questionnaire” and “somatic symptom scale 8”). This initial search employed no filters, and the full search terms are provided in the supplement. Second, we identified all records that cited the PHQ- 15 and SSS- 8 primary publications [10, 11]. Third, we searched specifically for clinical trials that could be useful for the evaluation of sensitivity to change (see the primary publication [9]). The final updated search was conducted on February 1, 2024. The systematic review included publications reporting a wide range of measurement properties including factor analysis, taxometric analysis, internal consistency, correlations relevant for construct validity, mean scores from general population or clinical samples, receiver operating characteristics, minimal clinically important difference, test–retest reliability, or sensitivity to change. Factor analyses were included regardless of sampling strategy, setting, and whether certain items had to be dropped in order to reach an adequate factor solution (common for the PHQ- 15). The present study focused on structural equation modeling strains of factor analysis because this was necessary for the model to reflect both a general somatic symptom burden factor and a series of more local factors at the same time.

### Extraction of Factor Analytic Data

Information from the articles was tabulated by JH and EA, with a reliability check based on a random subset of 20 studies. Extracted variables included author, year, country, language, study setting, and method of factor analysis (e.g., fit indices and estimators). The original study authors were contacted to ensure that the same study was not tabulated twice. Non-reported estimates were not requested. Quality assessment was conducted using a study-specific instrument based loosely on that described by Reilly and colleagues [18]. The criteria of this instrument relevant for the present study were investigator bias (Did the authors co-author the primary publication for the PHQ- 15 or SSS- 8?), sample size (Was the sample size at least 300?; crude rule of thumb based on published simulations Guadagnoli and Velicer [19, 20]), and sampling method (Was the focus on a general population or specific psychiatric diagnosis estimates, but without adequate sampling methods?).

### Synthesis and Statistical Analysis

The present study synthesized factor analyses that captured both a general somatic symptom burden factor and local symptom-domain factors; the latter typically standing for cardiopulmonary, fatigue, gastrointestinal, and pain symptoms. We reviewed bifactor models, i.e., models in which the general factor stood free from local domain factors (most common for the PHQ- 15), and also hierarchical models, i.e., models in which the domain factors loaded on an overarching general factor (most common for the SSS- 8). For each factor analysis, we tabulated the factor loadings of the specific symptoms on the general somatic symptom burden factor. For the hierarchical models, we also tabulated the factor loadings of the domain-specific factors onto the second-tier general somatic symptom burden factor. To avoid a false impression of precision due to notable heterogeneity across samples, questionnaire translations, and model specifications, we did not pool the factor loadings using meta-analytic methods. The distribution in factor loadings over original studies was instead summarized based on the mean and median, and we highlighted the three highest, and the one lowest, factor loadings from each factor analysis. To further elucidate the characteristics of the general and domain-specific factors, we also tabulated all reported correlates of the general and domain-specific factors. In the discussion of strengths of correlations, we abided by the guidelines of Cohen [21], according to which 0.10 can be regarded as small, 0.30 as moderate, and 0.50 as large.

## Results

### Overall Study Characteristics

The systematic review as a whole included 301 publications with 361,243 participants [9]. As previously reported, for the PHQ- 15, a bifactorial model was found to be preferable in 13/16 studies in which such a model was evaluated. For the SSS- 8, a hierarchical model was found to be preferable or equal to other alternatives in 4/5 studies. Typical factor structures are illustrated in Figure S1, but small deviations were common and especially for the PHQ- 15. As previously reported, the general factor explained a moderate proportion of the total variance, with AVEs of 0.19–0.45 for the PHQ- 15, and 0.30–0.51 for the SSS- 8.

The present study focused on factor analyses that allowed for a combination of a general somatic symptom burden factor and local symptom-domain factors, in terms of either a bifactor or hierarchical structure (Figure S2). We identified 23 such factor analyses, reported in 17 publications. All these publications were from 2013 or later, and we extracted data from 24,717 participants, approximately 61% women (around 15,114), with a combined mean age of 41 years (SD = 18; as based on 21/23 analyses). Out of the 23 identified factor analyses, 18 concerned the PHQ- 15 and 5 the SSS- 8. More characteristics are presented in Table 1.

**Table 1:** Studies included in the systematic review that reported a factor analysis that combined a general factor with local domain factors

Study reference	ID	Sample	Language	*N*	FA type	Models evaluated	Preferred
*PHQ-15*							
Cano-Garcia et al. (2020) [49]	1	Primary care, mental h	Spanish	1255	ESEM	1f, 4f, bf	bf
Leonhart et al. (2018) [31]	2	Psychosomatic clinic	German	2517	CFA	bf	bf
Leonhart et al. (2018) [31]	3	General population	Chinese	1329	CFA	bf	bf
Leonhart et al. (2018) [31]	4	Mixed	Dutch	456	CFA	bf	bf
Schlechter et al. (2023) [22]	5	Other/convenience	Arabic	182	CFA	1f, 3f, 4f, bf, hi	4f
Schlechter et al. (2023) [22]	6	Other/convenience	Arabic	187	CFA	1f, 3f, 4f, bf, hi	4f
Stauder et al. (2021) [50]	7	Other/convenience	Hungarian	5020	ESEM	1f, bf	bf
Taimeh et al. (2024) [23]	8	Other medical care	English	129	CFA	1f, 3f, 4f, bf	4f
Terluin et al. (2022) [48]	9	Mixed	Dutch	234	CFA	1f, bf	bf
Walentynowicz et al. (2018) [47]	10	Students	Dutch	1052	CFA	1f, 4f, hi, bf	bf
Witthöft et al. (2013) [16]	11	General population	German	414	EFA, CFA	1f, 4f, hi, bf	bf
Witthöft et al. (2013) [16]	12	Primary care, general	German	308	CFA	1f, 4f, hi, bf	bf
Witthöft et al. (2016) [24]	13	Students	German	1520	CFA	1f, hi, bf	bf
Witthöft et al. (2016) [24]	14	Students	German	3053	CFA	1f, hi, bf	bf
Zhang et al. (2016) [25]	15	Other medical care	Chinese	1329	EFA, CFA	1f, 3f, hi	3f
Zhou et al. (2020) [26]	16	Students	German	416	CFA	1f, hi	hi
Zhou et al. (2020) [26]	17	Students	Chinese	413	CFA	1f, hi	hi
Zolotareva (2023) [30]	18	General population	Russian	1153	EFA, CFA	1f, 3f, bf	bf
*SSS-8*							
Ghapanch et al. (2022) [51]	19	Mixed	Persian	122	CFA	1f, hi	unclear
Gierk et al. (2014) [11]	20	General population	German	2510	CFA	1f, hi	hi
Goodarzi et al. (2020) [29]	21	Other/convenience	Persian	281	CFA	hi	hi
Li et al. (2022) [27]	22	Other medical care	Chinese	699	CFA	1f, 3f, hi	3f
Petrelis and Domeyer (2022) [28]	23	Other medical care	Greek	138	CFA	hi	hi

See the original articles for detailed specifications which differed somewhat, including within studies. Note also that when articles reported several factor analyses (substudies), these are reported on separate lines

*Abbreviations*: *bf*, a bifactor model with symptom domain-specific factors and a separate general somatic symptom burden factor; *CFA*, confirmatory factor analysis; *EFA*, exploratory factor analysis; *ESEM*, exploratory structural equation modeling; *FA*, factor analysis; *hi*, a hierarchical model with symptom-specific factors and a second-order general somatic symptom burden factor

### Factor Loadings of Individual Symptoms on General Somatic Symptom Burden

Out of the 18 factor analyses focusing on the PHQ- 15, two did not converge (both reported by Schlechter et al. [22]) and five were reported without item factor loadings [23–26]. Factor loadings on the general factor in the 11 remaining analyses — all based on bifactor models — are presented in Table 2, with fit indices in Table S1. Among items included in most factor analyses, the highest mean and median factor loadings were seen for fatigue (tired or low energy; *M* = 0.65, median = 0.65) and dizziness (*M* = 0.63, median = 0.62). The fainting spells item was reportedly included in four factor analyses, in which its mean loading was 0.66 (median = 0.63). There were small differences in mean factor loadings among the top candidates. The item that had a top-three factor loading in most analyses was fatigue (tired or low energy; 9 out of 11 analyses), followed by dizziness (6/11) and chest pain (6/11). The lowest mean and median factor loading were reported for sexual pain/problems (*M* = 0.38, median = 0.39). This item also had the lowest loading in most factor analyses (5 out of 10).

**Table 2:** Factor loadings for specific symptoms on general somatic symptom burden

	Factor analysis identifier (see Table 1)											Summary statistics					
	1	2	3	4	7	9	10	11	12	13	18	*k*	*M*	SD	Median	In top 3	Lowest
*PHQ- 15*																	
1. Stomach pain	0.53	0.47	**0.59**	0.45	0.57	0.49	0.40	0.43	0.47	0.47	0.51	11	0.49	0.06	0.47	1	0
2. Back pain	**0.57**	0.57	0.53	0.57	0.64	0.49	0.33	0.49	0.40	*0.31*	0.53	11	0.49	0.11	0.53	1	1
3. Pain arms, legs, joints	0.49	0.51	0.47	**0.68**	0.63	0.65	0.27	0.46	*0.32*	*0.31*	0.48	11	0.48	0.14	0.48	1	2
4. Menstrual problems	-	-	-	-	-	-	-	-	-	-	-	0	-	-	-	-	-
5. Headaches	**0.64**	0.58	**0.87**	0.50	**0.74**	0.57	0.51	0.46	0.51	0.44	0.51	11	0.58	0.13	0.51	2	0
6. Chest pain	**0.57**	**0.62**	**0.59**	**0.81**	**0.75**	0.63	0.40	**0.71**	0.51	0.50	*0.45*	11	0.59	0.13	0.59	6	1
7. Dizziness	0.56	**0.68**	**0.71**	0.61	**0.74**	**0.70**	0.50	0.62	**0.70**	**0.54**	**0.59**	11	0.63	0.08	0.62	6	0
8. Fainting spells	-	0.58	0.53	0.67	-	**0.87**	-	-	-	-	-	4	0.66	0.15	0.63	1	0
9. Palpitations	0.53	-	-	-	**0.79**	0.67	0.40	0.68	**0.62**	0.51	0.53	8	0.59	0.12	0.58	2	0
10. Short breath	0.53	**0.59**	*0.42*	0.61	0.53	0.53	0.38	**0.70**	0.56	**0.70**	0.51	11	0.55	0.10	0.53	3	1
11. Sexual pain/problems	*0.20*	*0.38*	0.44	0.40	*0.50*	*0.37*	*0.32*	0.40	0.43	0.38	-	10	0.38	0.08	0.39	0	5
12. Constipation, diarrhea	0.43	0.47	*0.42*	0.40	0.53	**0.71**	0.34	*0.38*	0.43	0.48	0.46	11	0.46	0.10	0.43	1	2
13. Nausea, gas, dyspepsia	0.55	0.55	0.50	*0.39*	0.62	0.66	**0.54**	0.45	0.56	0.40	0.55	11	0.52	0.08	0.55	1	1
14. Tired or low energy	**0.65**	**0.59**	0.49	**0.76**	0.67	**0.70**	**0.65**	**0.63**	**0.75**	**0.53**	**0.72**	11	0.65	0.09	0.65	9	0
15. Trouble sleeping	0.54	0.51	0.47	0.48	**0.74**	0.52	**0.52**	0.61	0.51	0.43	**0.60**	11	0.54	0.08	0.52	3	0
	Factor analysis identifier (see Table 1)																
	19	20	21														
*SSS- 8*																	
1. Gastrointestinal	**0.59**	0.64	*0.40*									3	0.54	0.13	0.59	1	1
2. Back pain	0.54	0.68	**0.59**									3	0.60	0.07	0.59	1	0
3. Pain arms, legs, joints	0.56	0.71	**0.57**									3	0.61	0.08	0.57	1	0
4. Headaches	**0.64**	*0.52*	0.51									3	0.56	0.07	0.52	1	1
5. Chest pain, short breath	**0.67**	**0.81**	**0.60**									3	0.69	0.11	0.67	3	0
6. Dizziness	*0.44*	**0.80**	0.53									3	0.59	0.19	0.53	1	1
7. Tired or low energy	0.50	**0.78**	**0.57**									3	0.62	0.15	0.57	2	0
8. Trouble sleeping	*0.44*	0.72	0.46									3	0.54	0.16	0.46	0	1

For each factor analysis, the three highest factor loadings are in bold, and the lowest factor loading is in italics. Whenever a study presented both bifactor and hierarchical models, estimates from the model that had the best fit were tabulated. Dashed cells indicate that the corresponding item was not included in the factor solution

*Abbreviations*: *PHQ- 15*, Patient Health Questionnaire 15; *SSS- 8*, Somatic Symptom Scale 8

Out of the five factor analyses focusing on the SSS- 8, two were not reported with item factor loadings [27, 28]. Factor loadings on the general factor in the three other analyses — all based on hierarchical models — are presented in Table 2, with fit indices in Table S1. The highest mean factor loadings were seen for chest pain and shortness of breath (0.69), followed by fatigue (tired or low energy, 0.62). The highest median was seen for chest pain and shortness of breath (0.67), followed by gastrointestinal symptoms (0.59) and back pain (0.59). The items that had a top-three loading in most analyses were chest pain and shortness of breath (3 out of 3 analyses), followed by fatigue (tired or low energy; 2 out of 3 analyses). No SSS- 8 item had the lowest loading in more than one factor analysis.

### Factor Loadings of Domain-Specific Factors on General Somatic Symptom Burden

No publication reported estimates that made it possible to determine the loadings of domain-specific factors — e.g., cardiopulmonary, fatigue, gastrointestinal, and pain — on a general somatic symptom burden factor in a hierarchical model focusing on the PHQ- 15. There were three factor analyses that reported such estimates for the SSS- 8. These are reported in Table S2, which also includes the loadings of the single gastrointestinal item, as this loaded directly onto the general factor. The highest loadings, all above 0.90, were seen for the cardiopulmonary factor. The lowest loadings, all below 0.70, were seen for the gastrointestinal item.

### Correlates of General Somatic Symptom Burden

Eleven factor analyses evaluated correlates of the general somatic symptom burden factor, typically by incorporating additional variables in the structural equation modeling framework. In two analyses, it was unclear whether weighted scoring mirroring the factor analyses was employed, and these estimates are therefore only reported in the supplement (Table S3; [29, 30]). The correlates of the nine remaining factor analyses, all focusing on the PHQ- 15, are presented in Table 3. The association with symptoms of depression or negative affectivity was evaluated in seven of these nine analyses, typically with a moderate to strong relationship. There were, however, two notable exceptions, both from the same publication, where this association was weak but still statistically significant [31]. Two analyses, which were derived from one publication, concerned associations with variables indicative of a preoccupation with health or symptoms, namely health anxiety and somatosensory amplification [24]. These correlations were strong. The relationship was distinct from, but weaker than, that with depression symptoms when this was included as a competing predictor in the same regression model. Two studies reported correlations with functional somatic syndromes. The correlation with irritable bowel syndrome was moderate, and that with the presence of a functional somatic syndrome of any kind (broad definition; any out of 17) was moderate to strong [16, 24].

**Table 3:** Variables correlating with general somatic symptom burden and local symptom-domain factors

ID	Reference	Patient Health Questionnaire 15 (PHQ- 15)	
		Correlations with general somatic symptom burden factor	Correlations with local symptom-domain factors
2	Leonhart et al. (2018) [31]	Depression (PHQ- 9) std regression coefficient: 0.35 (*p* < 0.001) Anxiety (GAD- 7) std regression coefficient: 0.43 (*p* < 0.001)	Depression: 0.76 for fatigue, 0.03–0.09 for other local factors. Anxiety: 0.89 for fatigue, 0.02–0.09 for other local factors
3	Leonhart et al. (2018) [31]	Depression (PHQ- 9) std regression coefficient: 0.17 (*p* < 0.001) Anxiety (GAD- 7) std regression coefficient: 0.21 (*p* < 0.001)	Depression: 0.40 for fatigue, 0.08–0.21 for other local factors Anxiety: 0.38 for fatigue, 0.03–0.11 for other local factors
4	Leonhart et al. (2018) [31]	Depression (PHQ- 9) std regression coefficient: 0.18 (*p* = 0.002) Anxiety (GAD- 7) std regression coefficient: 0.17 (*p* = 0.006)	Depression: 0.04 for fatigue, 0.00–0.07 for other local factors Anxiety: 0.00 for fatigue, 0.01–0.06 for other local factors
7	Stauder et al. (2021) [50]	Depression (BDI-R) correlation: 0.66 (*p* < 0.001) Wellbeing (WHO- 5) correlation: − 0.58 (*p* < 0.001)	Depression: − 0.17 to 0.01 with the local factors Wellbeing: − 0.21 to − 0.07 with the local factors
9	Terluin et al. (2022) [48]	Correlation of 0.99 with another general somatic symptom burden factor derived from the Four-Dimensional Symptom Questionnaire (4DSQ-S)	-
10	Walentynowicz et al. (2018) [47]	Negative affectivity (PANAS) correlation: 0.53 (*p* < 0.001)	Negative affectivity: − 0.01 to 0.14 with the local factors
12	Witthöft et al. (2013) [16]	Irritable bowel syndrome (IBS) std regression coefficient: 0.38 (sign.)	IBS: 0.65 for gastro, − 0.03 to 0.14 for other local factors
13	Witthöft et al. (2016) [24]	Health anxiety (WI- 14) correlation: 0.62 (*p* < 0.001). When health anxiety and depression (7 distinct items of the PHQ- 9) were predictors in the same regression model, the std coefficient for health anxiety was 0.41 (*p* < 0.001) and the std coefficient for depression was 0.54 (*p* < 0.001)	Health anxiety: 0.07–0.26 with the local factors
14	Witthöft et al. (2016) [24]	Somatosensory amplification (SSAS) correlation: 0.53 (*p* < 0.001). When somatosensory amplification and depression (7 distinct items of the PHQ- 9) were predictors in the same regression model, the std coefficient for somatosensory amplification was 0.40 (*p* < 0.001) and the std coefficient for depression was 0.56 (*p* < 0.001) Functional somatic syndrome (broad definition; any out of 17, surveyed using the FFSS) std regression coefficient: 0.49 (*p* < 0.001)	Somatosensory amplification: 0.07–0.09 with the local factors Functional somatic syndrome 0.18 for gastro IBS: 0.83 for gastro

*Abbreviations*: *BDI-R*, Hungarian short version of the Beck Depression Inventory; *FFSS*, Questionnaire for the Assessment of Functional Somatic Syndromes; *PANAS*, Positive and Negative Affect Schedule; *PHQ- 9*, Patient Health Questionnaire 9; *std*, standardized; *SSAS*, Somatosensory Amplification Scale; *WHO- 5*, World Health Organization Five Well-Being Index; *WI- 14*, 14-item Whiteley Index

### Indicators of Risk of Bias

As was previously reported, the inter-rater reliability of the study-specific risk of bias instrument was nearly perfect (*κ* = 0.95–1.00) [9]. Concerning publications included in the present study, the risk of bias was deemed low with respect to investigator bias (16/17 publications, high in 1/17), mixed for sampling method (low in 2/17, high in 4/17, not applicable or unclear in 11/17), and mixed for sample size (low in 11/17, high in 6/17).

## Discussion

This study investigated general somatic symptom burden — the tendency to experience and report physical symptoms in general — based on published factor analyses of the PHQ- 15 and the SSS- 8. Taken together, this systematic review indicates that individuals who exhibit a generally high somatic symptom burden that cuts across symptom domains are especially likely to be bothered by cardiopulmonary symptoms and fatigue, and to report functional somatic syndromes and mental health problems. There was heterogeneity in the included studies, in terms of the exact specification of the factor structure, and to some extent regarding item inclusion for factor analysis. A general pattern yet appears to be that cardiopulmonary symptoms (e.g., dizziness, chest pain, and shortness of breath) and fatigue (i.e., feeling tired or lacking energy) are particularly strongly associated with the general factor. Typically, the general propensity for experiencing physical symptoms regardless of symptom domain also appears to be moderately to strongly associated with symptoms of depression, various forms of symptom preoccupation such as health anxiety, and functional somatic syndromes.

These results were largely in line with expectations. Though there are exceptions, we are aware of several original studies focusing on other scales than PHQ- 15 or SSS- 8 that have found cardiopulmonary symptoms and fatigue to be especially strongly associated with general somatic symptom burden [32–35]. Our findings are also in line with a widely referenced study by Fink and colleagues [14] who conducted a principal component analysis of medically unexplained symptoms reported by 978 internal medical, neurological, and primary care patients. In that study, the symptoms with the highest mean and median loadings on the general factor of the PHQ- 15 in the present study — fatigue and dizziness — did not load clearly onto any domain-specific factor. (Note here that principal component analysis does not allow for evaluation of a bifactor or hierarchical structure.) Moreover, in the same study, cluster analysis indicated that approximately 5% of patients belonged to a class that experienced a broad somatic symptom burden, with pronounced functional impairment. This class was found more closely related with general symptoms (including fatigue and dizziness) and various cardiopulmonary symptoms, rather than gastrointestinal and musculoskeletal symptoms. In a similar vein, researchers focusing specifically on the causes of fatigue have argued for this being regarded as a “generic response to illness.” For example, in a pooled cohort of 1696 patients with common chronic conditions, fatigue was found to be better predicted by transdiagnostic factors such as sex, age, motivational and concentration problems, pain, sleep disturbances, physical functioning, reduced activity, and lower self-efficacy concerning fatigue, than by specific medical conditions [15].

Overall, the findings presented here build on those presented previously [9] by indicating that not only does a general factor typically explain a moderate proportion of the overall variance in the PHQ- 15 and SSS- 8, but this general factor also appears to exhibit a reasonably stable pattern of associations with individual symptoms — notably cardiopulmonary symptoms and fatigue — and other constructs, that replicates over original studies. Witthöft et al. [16] suggested that the general factor of the PHQ- 15 could potentially represent the affective component of somatic symptom burden: something that could make the general factor a viable target of clinical intervention. This is also consistent with a large number of theoretical models delineating how psychological factors interact with physical complaints. However, the present study does not concern or elucidate the causes behind the observed associations between the general factor and individual somatic symptoms, or between the general factor and other variables. One possibility highlighted by previous authors is that because symptoms associated with stress, anxiety, and physiological arousal were strongly associated with general somatic symptom burden, persistent or heightened stress or anxiety may be a typical contributor to a general somatic symptom burden [14]. An analogous argument could be put forth for fatigue and depression. However, given the cross-sectional nature of these data, there are also other possible interpretations. Causality could be reversed, meaning that a general somatic symptom burden gives rise to poor mental health; something that has high face validity. Another possibility is that other variables — genetics and environmental factors — are the cause of both general somatic symptom burden and poor mental health. This view, that the propensity for a general somatic symptom burden shares risk and maintaining factors with internalizing psychopathology, is in line with recent work identifying these constructs as part of the same emotional dysfunction superspectrum [36]. One example of a study in support of such a hierarchical view of psychopathology included 5433 primary care patients from 14 countries and found that when depression symptoms, anxiety symptoms, and physical symptoms were included in the same factor analysis, the best fitting model had all items loading onto a single strong general internalizing factor, while a smaller proportion of the variance was explained by specific factors for anxiety, depression, and somatic complaints [37]. Moreover, twin studies of the genetic and environmental contribution to fatigue, pain, and functional somatic syndromes have typically found that genetic and environmental factors overlap with those underlying anxiety and depression [38–40]. An important caveat to these findings, however, is that a certain degree of overlap may exist solely by definition. For example, fatigue and sleep problems are often considered symptoms of depression (for example as criteria A4 and A6 for major depressive disorder according to the DSM- 5-TR [41]), and cardiopulmonary symptoms are often considered part of the experience of anxiety or fear. In summary, the causality underlying the subjective experience of physical symptoms is multifactorial and complex.

Much of the heterogeneity in factor loadings over studies tabulated here (Table 2) is probably related to the different sampling procedures. We deem it likely that this led to restriction of range effects in that when study participants (or patients) were selected on the basis of having a high level of distress and negative emotional responses to symptoms, this implied a selection on a high general somatic symptom burden factor (see above), which, as a mathematical necessity, reduced the strength of correlations with the general factor [42]. This suspicion is supported for example by a Danish study that contrasted the factor structure of the bodily distress syndrome (BDS) checklist — another symptom questionnaire, slightly more exhaustive than the PHQ- 15 or SSS- 8 — in the general population (*n*= 9656), primary care (*n*= 2480), and a specialist clinical setting focusing on functional somatic syndromes and psychosomatic complaints (*n*= 492) [43]. While the same bifactor model, similar to that typical of the PHQ- 15 (though also with a local factor for “general symptoms”), was preferred in all three samples, the factor loadings of items on the general factor were typically (though not always) lower in the specialist clinical setting data. For example, the loading for excessive fatigue on the general symptom burden factor dropped from 0.70 in the general population and primary care samples to 0.50 in the specialist sample [43]. Other probable key sources of heterogeneity include small variations in the specification of the factor structure, distinct cultural beliefs and practices in relation to psychological distress and somatic symptoms [44], the use of different translations, and the recruitment of different age groups [45] and socioeconomic strata [46].

### Strengths and Limitations

Important strengths of this study included the systematic and relatively exhaustive literature search, and that we were able to compare outcomes pertaining to the PHQ- 15 and SSS- 8. There were also limitations. First and foremost, we did not base our analyses on original data or meta-analytic methods. Instead, this review summarized estimates from each original article separately. This also meant that certain key estimates such as factor loadings could not be reviewed when these were not reported in the original journal articles. Notably, for this reason, factor loadings were also derived exclusively from bifactor models of the PHQ- 15, while loadings were from hierarchical models of the SSS- 8, and this may have affected the difference in outcome. Another aspect worth pointing out is that the extent to which physical symptoms share variance with other symptoms in a factor analysis is likely to depend on the precise selection of symptoms, and the emphasis on certain symptom domains over others in the item pool as a whole. This is not a limitation for conclusions about the PHQ- 15 or SSS- 8, but relevant for readers with an interest in wider generalization to the latent structure of somatic symptom burden in general. Speaking for some degree of external validity in this regard, similar factor structures have been seen for other instruments [e.g., 47, 48], and results focusing on cardiopulmonary symptoms and fatigue were in line with at least many of those presented based on other methods in several individual studies (see above). Last, another limitation is that this systematic review focused exclusively on peer-reviewed journal articles written in English, which implies that publications in other languages were systematically overlooked.

### Clinical and Research Implications

For clinicians, a take-home message from this study could be that patients who experience a general somatic symptom burden are especially likely to report high levels of fatigue and cardiopulmonary symptoms, and heightened indicators of poor mental health. This study says nothing about the reasons for this. For researchers with an interest in behavioral medicine and adjacent fields, a take-home message from this study could be that there is at least moderate consistency in the internal structure of the PHQ- 15, such that cardiopulmonary symptoms and fatigue appear to be especially closely associated with the general somatic symptom burden factor. Also, there appears to be a moderate to strong relationship between the general somatic symptom burden factor and markers of poor mental health, with especially strong evidence for depressive symptoms. Theoretically stringent longitudinal and experimental work is warranted for further clarification of the causal mechanisms behind the factor structure of the PHQ- 15.

### Conclusion

The general propensity for overall somatic symptom burden appears to be especially closely related with cardiopulmonary symptoms and fatigue, as opposed to other common physical symptoms. Further, the general propensity toward somatic symptom burden appears to be closely associated with indicators of poor mental health and functional somatic syndromes, and typically more so than the local, domain-specific factors. The reasons for this are likely to be complex and multifactorial. Further longitudinal and experimental studies are warranted to clarify the causality behind the indicated structure of somatic symptom burden.

## Supplementary Information

Below is the link to the electronic supplementary material.

Supplementary file1 (PDF 377 KB)

Supplementary file2 (PDF 487 KB)

## Data Availability

All data analyzed in this study are reported as part of the results.
