# Supplementary material for: Understanding General Somatic Symptom Burden: Insights from a Systematic Review of Factor Analyses Pertaining to the Patient Health Questionnaire 15 (PHQ-15) and Somatic Symptom Scale 8 (SSS-8)
Source: Int J Behav Med. 2025 Apr 23;33(2):264–73. doi: 10.1007/s12529-025-10365-y (PMC13160965; doi:10.1007/s12529-025-10365-y)
Supplement: Supplementary file 1 — Supplementary file1 (PDF 377 KB) [file 12529_2025_10365_MOESM1_ESM.pdf]

Understanding general somatic symptom burden: Supplementary material

Figure S1. *Typical factor solutions for the Patient Health Questionnaire 15 (PHQ-15) and Somatic Symptom Scale 8 (SSS-8).* Note that the factor structure for the PHQ-15 reported by Witthöft et al. (2013) [1] which is widely referenced across the literature did have the sexual pain/problems item loading onto the gastrointestinal factor, though this has not always been replicated (e.g., Leonhart et al., 2018 [2]).

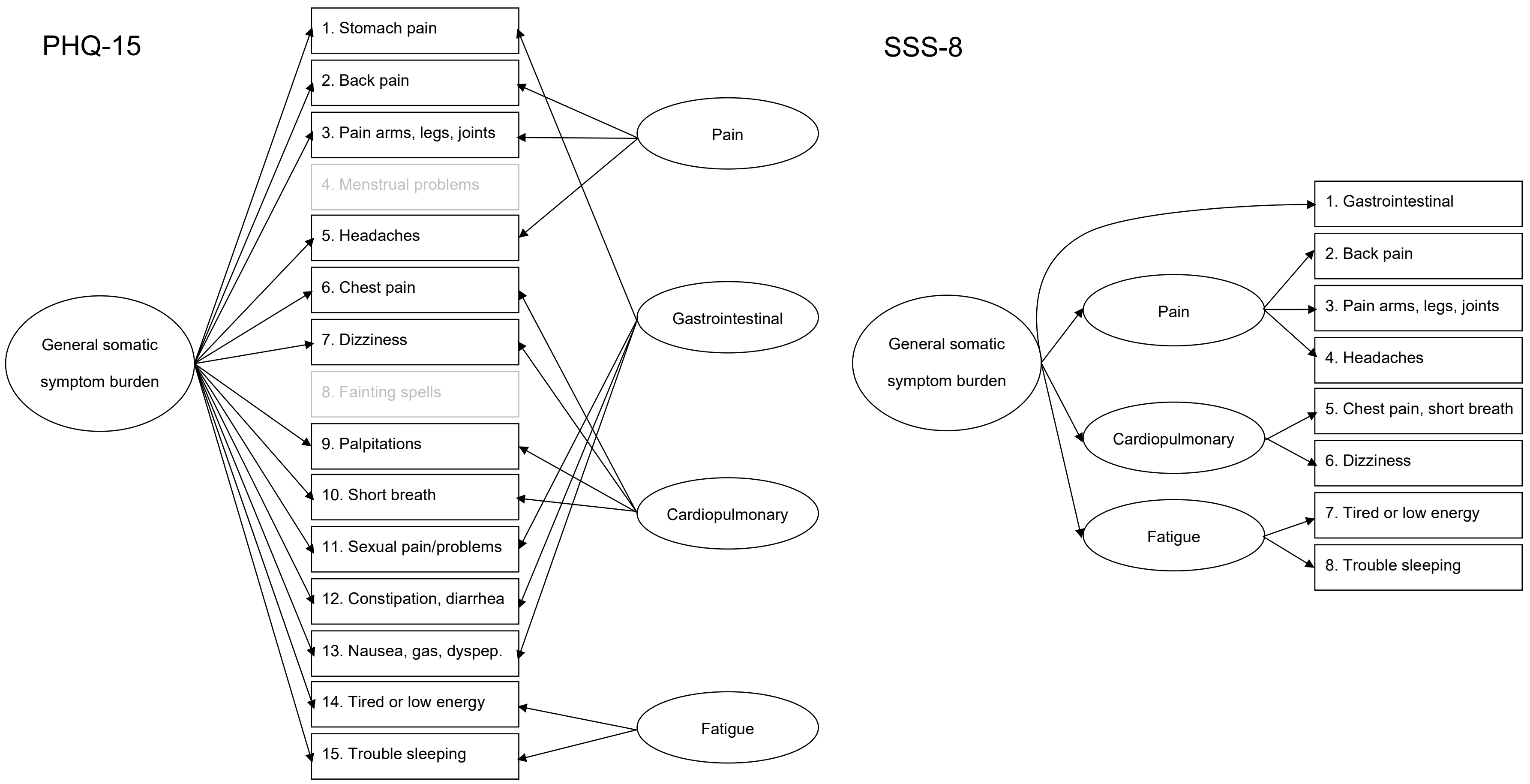

Figure S2. *Overview of study selection process*

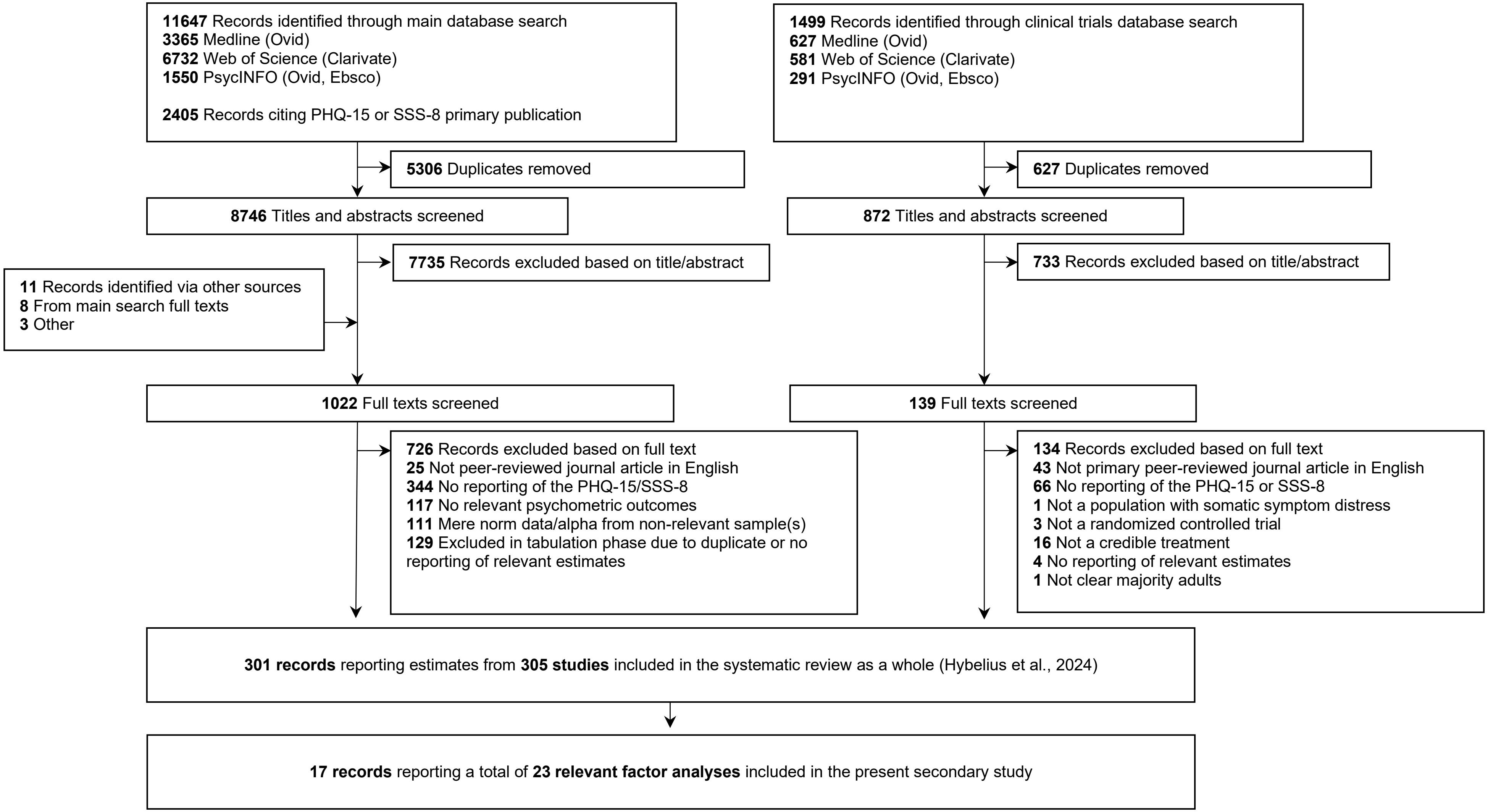

Table S1. *Fit indices for bifactor and hierarchical factor solutions from which factor loadings could be evaluated in this study*

Study reference	ID	Sample	Language	N	FA type	Type	CFI	TLI	RMSEA
PHQ-15									
Cano-Garcia et al. (2020) [3]	1	Primary Care, Mental H.	Spanish	1255	ESEM	bf	1.00	1.00	0.01
Leonhart et al. (2018) [2]	2	Psychosomatic Clinic	German	2517	CFA	bf	0.98	0.97	0.044
Leonhart et al. (2018) [2]	3	General Population	Chinese	1329	CFA	bf	0.98	0.96	0.052
Leonhart et al. (2018) [2]	4	Mixed	Dutch	456	CFA	bf	0.998	0.997	0.019
Stauder et al. (2021) [4]	7	Other/Convenience	Hungarian	5020	ESEM	bf	0.99	0.98	0.037
Terluin et al. (2022) [5]	9	Mixed	Dutch	234	CFA	bf	0.97	0.98	0.052
Walentynowicz et al. (2018) [6]	10	Students	Dutch	1052	CFA	bf	0.99		0.019
Witthöft et al. (2013) [1]	11	General Population	German	414	EFA, CFA	bf	0.98	0.97	0.040
Witthöft et al. (2013) [1]	12	Primary Care, General	German	308	CFA	bf	0.97	0.95	0.053
Witthöft et al. (2016) [7]	13	Students	German	1520	CFA	bf	0.992	0.99	0.020
Zolotareva (2023) [8]	18	General Population	Russian	1153	EFA, CFA	bf	0.97	0.96	0.044
SSS-8									
Ghapanch et al. (2022) [9]	19	Mixed	Persian	122	CFA	hi	0.99		0.001
Gierk et al. (2014) [10]	20	General Population	German	2510	CFA	hi	0.97	0.95	0.08
Goodarzi et al. (2020) [11]	21	Other/Convenience	Persian	281	CFA	hi	0.96		0.069

Note. See the original articles for detailed specifications which differed somewhat, including within studies. Note also that when articles reported several factor analyses (substudies), these are reported on separate lines. Abbreviations: bf, a bifactor model with symptom domain-specific factors and a separate general somatic symptom burden factor; CFA, confirmatory factor analysis; EFA, exploratory factor analysis; ESEM, exploratory structural equation modeling; FA, factor analysis; hi, a hierarchical model with symptom-specific factors and a second-order general somatic symptom burden factor.

Table S2. *Factor loadings for domain-specific factors on general somatic symptom burden in hierarchical models*

	**Analysis identifier (see Table 1)**				**Summary statistics**			
	**19**	**20**	**21**		**k**	**M**	**SD**	**Median**
SSS-8								
Cardiopulmonary factor	0.91	0.93	0.99		3	0.94	0.04	0.93
Gastrointestinal item	0.59	0.64	0.40		3	0.54	0.13	0.59
Fatigue factor	0.66	0.88	0.82		3	0.79	0.11	0.82
Pain factor	0.83	0.85	0.91		3	0.86	0.04	0.85

Abbreviations: SSS-8, Somatic Symptom Scale 8.

Table S3. *Variables correlating with general somatic symptom burden and local symptom-domain factors*

ID	Reference	Patient Health Questionnaire 15 (PHQ-15)	
		**Correlations with general somatic symptom burden factor**	**Correlations with local symptom-domain factors**
18	Zolotareva (2003) [8] [a b]	Physical symptoms (SCL-90-R) correlation: 0.82 ($p < .001$)<br>Depression (SCL-90-R) correlation: 0.66 ($p < .001$)<br>Anxiety (SCL-90-R) correlation: 0.68 ($p < .001$)	Physical symptoms: 0.55-0.73 with the local factors.<br>Depression: 0.44-0.62 with the local factors.<br>Anxiety: 0.47-0.63 with the local factors.
		**Somatic Symptom Scale 8 (SSS-8)**	
		**Correlations with general somatic symptom burden factor**	**Correlations with local symptom-domain factors**
21	Goodarzi et al. (2020) [11] [b]	-	Anxiety (BAI): 0.56-0.59 with the local factors.<br>General health (GHQ): 0.47-0.59 with the local factors.

Abbreviations: BAI, Beck Anxiety Inventory; GHQ, General Health Qestionnaire; SCL-90-R, Symptom Checklist 90 Revised.

[a] Additional subscales of the SCL-90-R were also evaluated.

[b] Unclear if weighted scoring was employed.

References

1. Witthöft M, Hiller W, Loch N, Jasper F. The latent structure of medically unexplained symptoms and its relation to functional somatic syndromes. *Int J Behav Med*. 2013;20(2):172-83.

2. Leonhart R, de Vroege L, Zhang L, Liu Y, Dong Z, Schaefert R, et al. Comparison of the Factor Structure of the Patient Health Questionnaire for Somatic Symptoms (PHQ-15) in Germany, the Netherlands, and China. A Transcultural Structural Equation Modeling (SEM) Study. *Front Psychiatry*. 2018;9:240.

3. Cano-Garcia FJ, Munoz-Navarro R, Sese Abad A, Moretti LS, Medrano LA, Ruiz-Rodriguez P, et al. Latent structure and factor invariance of somatic symptoms in the patient health questionnaire (PHQ-15). *J Affect Disord*. 2020;261:21-9.

4. Stauder A, Witthöft M, Köteles F. Validation of the Hungarian PHQ-15. A latent variable approach. *Ideggyogy Sz*. 2021;74(5-6):183-90.

5. Terluin B, Barends H, van der Horst HE, Dekker J, van der Wouden JC. Head-to-head comparison of somatic symptom scales: The Patient Health Questionnaire (PHQ-15) and the somatization scale of the Four-Dimensional Symptom Questionnaire (4DSQ-S). *J Psychosom Res*. 2022;162:111031.

6. Walentynowicz M, Witthöft M, Raes F, van Diest I, van den Bergh O. Sensory and affective components of symptom perception. *Journal of Experimental Psychopathology*. 2018;9(2).

7. Witthöft M, Fischer S, Jasper F, Rist F, Nater UM. Clarifying the latent structure and correlates of somatic symptom distress: A bifactor model approach. *Psychol Assess*. 2016;28(1):109-15.

8. Zolotareva AA. Medically Unexplained Symptoms among Adults from Russia: An Assessment using the Patient Health Questionnaire-15. *Psychol Russ*. 2023;16(2):33-47.

9. Ghapanch A, Abasi I, Bitarafan M, Zarabi H, Derakhshan FS, Derakhshan MK, et al. The Psychometric Evaluation of Somatic Symptom Scale-8 in Patients With Major Depressive Disorder. *Practice in Clinical Psychology*. 2022;10(1):69-78.

10. Gierk B, Kohlmann S, Kroenke K, Spangenberg L, Zenger M, Brähler E, et al. The Somatic Symptom Scale-8 (SSS-8): A brief measure of somatic symptom burden. *JAMA Intern Med*. 2014;174(3):399-407.

11. Goodarzi M, Ahmadi SM, Asle Zaker Lighvan M, Rahmati F, Molavi R, Mohammadi M. Investigating the Psychometric Properties of the 8-Item Somatic Symptom Scale in Non-clinical Sample of Iranian People. *Practice in Clinical Psychology*. 2020:57-64.
