# Supplementary material for: Understanding General Somatic Symptom Burden: Insights from a Systematic Review of Factor Analyses Pertaining to the Patient Health Questionnaire 15 (PHQ-15) and Somatic Symptom Scale 8 (SSS-8)
Source: Int J Behav Med. 2025 Apr 23;33(2):264–73. doi: 10.1007/s12529-025-10365-y (PMC13160965; doi:10.1007/s12529-025-10365-y)
Supplement: Supplementary file 2 — Supplementary file2 (PDF 487 KB) [file 12529_2025_10365_MOESM2_ESM.pdf]

Documentation of search strategies
University Library search consultation group

Date: 1 February 2024

Topic/research question: PHQ-15 / SSS-8

Name of researcher(s): Erland Axelsson, Jonna Hybelius

Librarian(s): Emma-Lotta Säätelä

Databases:

1. Ovid (Medline)
2. Web of Science Core Collection (Clarivate)
3. Psycinfo (Ebsco)

Total number of hits

- Before deduplication: 11,647
- After deduplication: 3,069 (+ 4,626 from previous search)

Comments:

After the original search was performed on 9 October 2021, the search was last updated on 1 February 2024 using the methods described by Bramer et al (1).

De-duplication was done using the method described by Bramer et al (2). One final, extra step was added to compare DOIs.

References

1. Bramer W, Bain P. (2017). Updating search strategies for systematic reviews using EndNote. *Journal of the Medical Library Association: JMLA*, 105(3):285-289. doi: 10.5195/jmla.2017.183.

2. Bramer, W. M., Giustini, D., de Jonge, G. B., Holland, L., & Bekhuis, T. (2016). De-duplication of database search results for systematic reviews in EndNote. *Journal of the Medical Library Association: JMLA*, 104(3), 240-243. doi: 10.3163/1536-5050.104.3.014

1. Medline

Interface: Ovid MEDLINE(R) ALL<br><br>Date of Search: 1 February 2024<br><br>Number of hits: 3,365<br><br>Comment: In Ovid, two or more words are automatically searched as phrases; i.e. no quotation marks are needed	Field labels<br><br>• exp/ = exploded MeSH term<br>• / = non exploded MeSH term<br>• .ti,ab,kf. = title, abstract and author keywords<br>• adjx = within x words, regardless of order<br>• * = truncation of word for alternate endings

Database(s): **Ovid MEDLINE(R) ALL** 1946 to January 26, 2024
Search Strategy:

#	Searches	Results
1	exp Patient Health Questionnaire/	976
2	("patient health questionnaire*" or PHQ or "somatic symptom scale 8" or SSS-8).ti,ab,kf.	14321
3	1 or 2	14612
4	exp "Factor Analysis, Statistical"/ or exp Psychometrics/ or exp "Reproducibility of Results"/ or exp "Sensitivity and Specificity"/	1042064
5	(psychometric* or valid* or factor analy* or sensitivity or specificity or test-retest).ti,ab,kf.	2290510
6	4 or 5	2888177
7	3 and 6	3365

2. Web of Science Core Collection

Interface: Clarivate Analytics<br><br>Editions = A&HCI , ESCI , SCI-EXPANDED , SSCI<br><br>Date of Search: 1 February 2024<br><br>Number of hits: 6,732	Field labels<br><br>• TS/Topic = title, abstract, author keywords and Keywords Plus<br>• NEAR/x = within x words, regardless of order<br>• * = truncation of word for alternate endings<br><br>Note: the *Exact search*-function was used for all the searches

#	Search Query	Results
1	TS=("patient health questionnaire*" or PHQ or "somatic symptom scale 8" or SSS-8)	15839
2	TS=(psychometric* or valid* or "factor analy*" or sensitivity or specificity or "test-retest")	4033364
3	#2 AND #1	6732

3. Psycinfo

Interface: EBSCO<br><br>Date of Search: 1 February 2024<br><br>Number of hits: 1,550	Field labels<br><br>• DE = subject heading<br>• TI = title<br>• AB = abstract<br>• KW = author keywords<br>• Nx = within x words, regardless of order<br>• * = truncation of word for alternate endings<br><br>Note: the *Apply equivalent subjects*-function was used for all the searches

#	Query	Results
S5	S1 AND S4	1,550
S4	S2 OR S3	658,123
S3	TI (psychometric* or valid* or "factor analy*" or sensitivity or specificity or "test-retest") OR AB (psychometric* or valid* or "factor analy*" or sensitivity or specificity or "test-retest") OR KW (psychometric* or valid* or "factor analy*" or sensitivity or specificity or "test-retest")	499,081
S2	DE "Psychometrics" OR DE "Classical Test Theory" OR DE "Consistency (Measurement)" OR DE "Error of Measurement" OR DE "External Validity" OR DE "Factor Analysis" OR DE "Internal Validity" OR DE "Item Analysis (Test)" OR DE "Item Response Theory" OR DE "Measurement Invariance" OR DE "Measurement Models" OR DE "Multivariate Analysis" OR DE "Test Construction" OR DE "Test Reliability" OR DE "Test Sensitivity" OR DE "Test Specificity" OR DE "Test Validity" OR DE "Variability Measurement" OR DE "Factor Analysis" OR DE "Confirmatory Factor Analysis" OR DE "Exploratory Factor Analysis" OR DE "Factor Structure" OR DE "Item Analysis (Statistical)" OR DE "Statistical Rotation" OR DE "Item Analysis (Test)" OR DE "Differential Item Functioning" OR DE "Measurement Models" OR DE "Mixture Modeling" OR DE "Structural Equation Modeling" OR DE "Multivariate Analysis" OR DE "Factor Analysis" OR DE "Mixture Modeling" OR DE "Multiple Regression" OR DE "Path Analysis" OR DE "Principal Component Analysis" OR DE "Test Reliability" OR DE "Internal Consistency" OR DE "Interrater Reliability" OR DE "Split-Half Reliability" OR DE "Test-Retest Reliability" OR DE "Test Validity" OR DE "Clinical Validity" OR DE "Construct Validity" OR DE "Content Validity" OR DE "Criterion Validity" OR DE "Face Validity" OR DE "Factorial Validity" OR DE "Variability Measurement" OR DE "Analysis of Covariance" OR DE "Analysis of Variance" OR DE "Interaction Variance" OR DE "Standard Deviation" OR DE "Statistical Rotation" OR DE "Oblique Rotation" OR DE "Orthogonal Rotation" OR DE "Mixture Modeling" OR DE "Latent Class Analysis" OR DE "Latent Profile Analysis" OR DE "Structural Equation Modeling" OR DE "Latent Class Analysis" OR DE "Clinical Validity" OR DE "Discriminative Validity" OR DE "Test Responsiveness" OR DE "Construct Validity" OR DE "Convergent Validity" OR DE "Discriminant Validity" OR DE "Nomological Validity" OR DE "Criterion Validity" OR DE "Concurrent Validity" OR DE "Incremental Validity" OR DE "Predictive Validity"	327,271
S1	TI ("patient health questionnaire" or PHQ or "somatic symptom scale 8" or SSS-8) OR AB ("patient health questionnaire" or PHQ or "somatic symptom scale 8" or SSS-8) OR KW ("patient health questionnaire" or PHQ or "somatic symptom scale 8" or SSS-8)	3,125

Documentation of search strategies
University Library search consultation group

Date: 2 February 2024

Topic/research question: Sensitivity to change

Name of researcher(s): Erland Axelsson, Jonna Hybelius

Librarian(s): Emma-Lotta Säätelä

Databases:

4. Medline (Ovid)
5. Web of Science Core Collection (Clarivate)
6. Psycinfo (Ebsco)

Total number of hits:

- Before deduplication: 1,499
- After deduplication: 151 (+ 721 from previous search)

---

1. Medline

Interface: Ovid MEDLINE(R) ALL<br><br>Date of Search: 2 February 2024<br><br>Number of hits: 627<br><br>Comment: In Ovid, two or more words are automatically searched as phrases; i.e. no quotation marks are needed	Field labels<br><br>• exp/ = exploded MeSH term<br>• / = non exploded MeSH term<br>• .ti,ab,kf. = title, abstract and author keywords<br>• adjx = within x words, regardless of order<br>• * = truncation of word for alternate endings

Database(s): **Ovid MEDLINE(R) ALL** 1946 to February 01, 2024
Search Strategy:

#	Searches	Results
1	randomized controlled trial.pt.	607974
2	controlled clinical trial.pt.	95543
3	randomi?ed.ab.	756173
4	placebo.ab.	245414
5	clinical trials as topic.sh.	201704
6	randomly.ab.	426320
7	trial.ti.	302148
8	or/1-7	1625156
9	(systematic review or meta-analy*).ti.	330057
10	8 not 9	1528481
11	exp animals/ not humans.sh.	5193302
12	10 not 11	1402310
13	exp Patient Health Questionnaire/	979
14	("patient health questionnaire" or PHQ or "somatic symptom scale 8" or "8-item somatic symptom scale" or SSS-8 or somatization or "somatic symptom burden" or "somatic symptom severity" or "somatic symptom distress").ti,ab,kf.	19758
15	or/13-14	20046
16	12 and 15	2621
17	Behavioral Medicine/ or Psychosomatic Medicine/ or exp Somatoform Disorders/ or Medically Unexplained Symptoms/ or Burning Mouth Syndrome/ or exp Colonic Diseases, Functional/ or Dyspepsia/ or Fatigue Syndrome, Chronic/ or Fibromyalgia/ or Hyperventilation/ or Multiple Chemical Sensitivity/ or exp Pain/ or exp Premenstrual Syndrome/ or Temporomandibular Joint Dysfunction Syndrome/ or Tension-Type Headache/ or Whiplash Injuries/	546124
18	transdiagnostic.ti,ab,kf.	4653

19	(somatoform or somatiz* or "somatic symptom and related disorders" or "somatic symptom disorder" or SSD or "somatic symptom distress" or "persistent somatic symptoms" or "bodily distress" or "medically unexplained" or MUS or "functional somatic" or "burning mouth" or "irritable bowel syndrome" or IBS or dyspepsia or "chronic fatigue" or CFS or fibromyalgia or "globus syndrome" or hyperventilation or "multiple chemical" or (chronic adj3 pain) or (persistent adj3 pain) or "atypical chest pain" or "non-cardiac chest pain" or "premenstrual syndrome" or "pseudo-epileptic" or "temporomandibular joint dysfunction syndrome" or "tension headache" or whiplash or neurasthenia or conversion).ti,ab,kf.	468778
20	or/17-19	912211
21	16 and 20	763
22	limit 21 to yr="2000 -Current"	661
23	limit 22 to english language	627

2. Web of Science Core Collection

Interface: Clarivate Analytics<br><br>Editions = A&HCI , ESCI , SCI-EXPANDED , SSCI<br><br>Date of Search: 2 February 2024<br><br>Number of hits: 581	Field labels<br><br>• TS/Topic = title, abstract, author keywords and Keywords Plus<br>• NEAR/x = within x words, regardless of order<br>• * = truncation of word for alternate endings<br><br>Note: the *Exact search*-function was used for all the searches

#	Search Query	Results
1	TI=(”randomi$ed” OR ”randomi$ed” OR ”randomi$ation” OR ”randomi$ation” OR placebo* OR (random* AND (allocat* OR assign*)) OR (blind* AND (”single” OR ”double” OR ”treble” OR ”triple”))) OR AB=(”randomi$ed” OR ”randomi$ed” OR ”randomi$ation” OR ”randomi$ation” OR placebo* OR (random* AND (allocat* OR assign*)) OR (blind* AND (”single” OR ”double” OR ”treble” OR ”triple”)))	1154499
2	TI=(”systematic review” or ”meta-analy*”)	388018
3	#1 NOT #2	1057679
4	TS=("patient health questionnaire" OR PHQ OR "somatic symptom scale 8" OR "8-item somatic symptom scale" OR SSS-8 OR somatization OR "somatic symptom burden" OR "somatic symptom severity" OR "somatic symptom distress")	24103
5	#3 AND #4	2358
6	TS=(somatoform OR somatiz* OR "somatic symptom and related disorders" OR "somatic symptom disorder" OR SSD OR "somatic symptom distress" OR "persistent somatic symptoms" OR "bodily distress" OR "medically unexplained" OR MUS OR "functional somatic" OR "burning mouth" OR "irritable bowel syndrome" OR IBS OR dyspepsia OR "chronic fatigue" OR CFS OR fibromyalgia OR "globus syndrome" OR hyperventilation OR "multiple chemical" OR (chronic NEAR/2 pain) OR (persistent NEAR/2 pain) OR "atypical chest pain" OR "non-cardiac chest pain" OR "premenstrual syndrome" OR "pseudo-epileptic" OR "temporomandibular joint dysfunction syndrome" OR "tension headache" OR whiplash OR neurasthenia OR conversion OR transdiagnostic)	1057316
7	#5 AND #6 and Proceeding Paper or Meeting Abstract (Exclude – Document Types) Timespan: 2000-01-01 to 2024-12-31	581

3. Psycinfo

Interface: EBSCO<br><br>Date of Search: 2 February 2024<br><br>Number of hits: 291	Field labels<br><br>• DE = subject heading<br>• TI = title<br>• AB = abstract<br>• KW = author keywords<br>• Nx = within x words, regardless of order<br>• * = truncation of word for alternate endings<br><br>Note: the *Apply equivalent subjects*-function was used for all the searches

#	Query	Results
S15	S10 AND S14<br>Limiters - Publication Date: 20000101-20240131<br>Source type: Academic journals	291
S14	S11 OR S12 OR S13	115,446
S13	TI (somatoform OR somatiz* OR "somatic symptom and related disorders" OR "somatic symptom disorder" OR SSD OR "somatic symptom distress" OR "persistent somatic symptoms" OR "bodily distress" OR "medically unexplained" OR MUS OR "functional somatic" OR "burning mouth" OR "irritable bowel syndrome" OR IBS OR dyspepsia OR "chronic fatigue" OR CFS OR fibromyalgia OR "globus syndrome" OR hyperventilation OR "multiple chemical" OR (chronic N2 pain) OR (persistent N2 pain) OR "atypical chest pain" OR "non-cardiac chest pain" OR "premenstrual syndrome" OR "pseudo-epileptic" OR "temporomandibular joint dysfunction syndrome" OR "tension headache" OR whiplash OR neurasthenia OR conversion) OR AB (somatoform OR somatiz* OR "somatic symptom and related disorders" OR "somatic symptom disorder" OR SSD OR "somatic symptom distress" OR "persistent somatic symptoms" OR "bodily distress" OR "medically unexplained" OR MUS OR "functional somatic" OR "burning mouth" OR "irritable bowel syndrome" OR IBS OR dyspepsia OR "chronic fatigue" OR CFS OR fibromyalgia OR "globus syndrome" OR hyperventilation OR "multiple chemical" OR (chronic N2 pain) OR (persistent N2 pain) OR "atypical chest pain" OR "non-cardiac chest pain" OR "premenstrual syndrome" OR "pseudo-epileptic" OR "temporomandibular joint dysfunction syndrome" OR "tension headache" OR whiplash OR neurasthenia OR conversion) OR KW (somatoform OR somatiz* OR "somatic symptom and related disorders" OR "somatic symptom disorder" OR SSD OR "somatic symptom distress" OR "persistent somatic symptoms" OR "bodily distress" OR "medically unexplained" OR MUS OR "functional somatic" OR "burning mouth" OR "irritable bowel syndrome" OR IBS OR dyspepsia OR "chronic fatigue" OR CFS OR fibromyalgia OR "globus syndrome" OR hyperventilation OR "multiple chemical" OR (chronic N2 pain) OR (persistent N2 pain) OR "atypical chest pain" OR "non-cardiac chest pain" OR "premenstrual syndrome" OR "pseudo-epileptic" OR "temporomandibular joint dysfunction syndrome" OR "tension headache" OR whiplash OR neurasthenia OR conversion)	61,842
S12	TI transdiagnostic OR AB transdiagnostic OR KW transdiagnostic	4,990
S11	DE ("Behavioral Medicine" OR "Psychosomatic Medicine" OR "Somatoform Disorders" OR "Conversion Disorder" OR "Factitious Disorders" OR "Neurasthenia" OR "Somatization Disorder" OR "Somatoform Pain Disorder" OR "Irritable Bowel Syndrome" OR Dyspepsia OR "Premenstrual Syndrome" OR	70,807

	Pain OR "Hyperventilation syndrome" OR "Chronic Fatigue Syndrome" OR "Muscle Contraction Headache" OR Whiplash)	
S10	S8 AND S9	747
S9	TI ("patient health questionnaire" OR PHQ OR "somatic symptom scale 8" OR "somatic symptom scale-8" OR "8-item somatic symptom scale" OR SSS-8 OR somatization OR "somatic symptom burden" OR "somatic symptom severity" OR "somatic symptom distress") OR AB ("patient health questionnaire" OR PHQ OR "somatic symptom scale 8" OR "somatic symptom scale-8" OR "8-item somatic symptom scale" OR SSS-8 OR somatization OR "somatic symptom burden" OR "somatic symptom severity" OR "somatic symptom distress") OR KW ("patient health questionnaire" OR PHQ OR "somatic symptom scale 8" OR "somatic symptom scale-8" OR "8-item somatic symptom scale" OR SSS-8 OR somatization OR "somatic symptom burden" OR "somatic symptom severity" OR "somatic symptom distress")	9,198
S8	S6 NOT S7	205,932
S7	TI (”systematic review” OR meta-analy*).	56,925
S6	S1 OR S2 OR S3 OR S4 OR S5	216,888
S5	TI trial	49,565
S4	AB randomly	85,634
S3	AB placebo	44,159
S2	AB randomi?ed	101,511
S1	DE ”Randomized Controlled Trials”	1,048
